# A culturally tailored Zhuang embroidery intervention for psychological well-being among Zhuang adults in the retirement transition in Guangxi: a waitlist-controlled quasi-experimental study

**DOI:** 10.3389/fpubh.2026.1945265

**Published:** 2026-09-17

**Authors:** Junfeng Li, Junzi Xiang

**Affiliations:** School of Fine Arts and Design, Yulin Normal University, Yulin, Guangxi, China

**Keywords:** cultural adaptation, healthy aging, minority arts, psychological well-being, retirement transition, waitlist-controlled quasi-experiment, Zhuang embroidery

## Abstract

**Background:**

Arts- and craft-based community programs may support psychological well-being in later life, yet few studies have examined culturally tailored ethnic heritage crafts as public health interventions in their communities of origin.

**Methods:**

This waitlist-controlled quasi-experimental study evaluated an eight-week intervention integrating Zhuang embroidery practice, cultural storytelling, and group exchange among community-dwelling Zhuang adults aged 55–65 years in Guangxi, China. Eighty participants were allocated by community cluster to intervention or waitlist control groups and assessed at baseline, immediately post-intervention, and 12-week follow-up. Psychological well-being was the primary outcome; loneliness, social connectedness, general well-being, ethnic identity, process perception, and qualitative interview findings were secondary or exploratory materials.

**Results:**

The intervention group showed greater immediate improvement in Ryff psychological well-being than waitlist controls. Baseline-adjusted sensitivity analysis supported the immediate Ryff signal, whereas the follow-up difference was attenuated. Secondary outcomes were less consistent: WEMWBS and loneliness showed short-term favorable movement, whereas social connectedness, WHO-5, and ethnic identity findings were weaker or exploratory. Process ratings and interviews suggested that participants recognized the cultural storytelling, needlework, and group exchange components.

**Conclusion:**

The integrated Zhuang embroidery intervention appears feasible and shows preliminary promise for psychological well-being among retirement-transition Zhuang adults. Because allocation occurred at the cluster level and the intervention combined embroidery practice, cultural storytelling, and group exchange, the findings cannot separate intervention-related signals from community differences, expectancy effects, or component-specific effects. Larger multi-community and component-separated studies with longer follow-up are needed before drawing confirmatory efficacy or mechanism conclusions.

## 1 Introduction

As populations continue to age, maintaining psychological well-being, social engagement, and a sense of life purpose alongside increased longevity has emerged as a pressing concern in public health and healthy aging research. Psychological well-being extends well beyond positive affect or life satisfaction; rather, it encompasses more developmental dimensions of psychological resources, including sense of life purpose, self-acceptance, positive relationships, environmental mastery, and personal growth (1). The retirement transition represents a significant life event within the aging process, typically involving withdrawal from occupational roles, reorganization of daily routines, shifts in social networks, and reconstruction of personal identity. While retirement does not inevitably lead to loneliness or psychological distress, recent longitudinal findings paint a more nuanced picture: the relationship between retirement and outcomes such as loneliness, social isolation, and well-being is contingent upon multiple factors, including the nature of the retirement transition, availability of social support, cultural context, and opportunities for social participation (2–7). From a public health perspective, the retirement transition should not be dismissed as a period of vulnerability or pathology; instead, it merits recognition as a time of continued functional capacity that nonetheless benefits from adequate social connection and psychological support, a critical window for preventive intervention. Within this window, loneliness and social connectedness emerge as particularly salient outcomes: loneliness reflects individuals’ subjective perception of insufficient social relationships, while social connectedness captures the quality of connections one maintains with others, groups, and communities. Existing reviews and empirical studies document strong associations between loneliness, social isolation, social disconnectedness, and psychological health risks in older populations; though intervention evidence continues to accumulate, effect heterogeneity remains substantial, and group-based activities, skill-building programs, social support, and connectedness-focused approaches show promise yet are often contingent upon implementation context and sustained participation (8–13). This suggests that interventions targeting retirement-transition populations must move beyond simply increasing contact opportunities; rather, they need activity formats that spark sustained participation, foster shared experience, and cultivate a renewed sense of life purpose.

Arts engagement, craft activities, and cultural participation offer promising pathways for promoting mental health in community-dwelling older adults. Systematic reviews, randomized trials, and arts-based prescription research suggest that group art-making, visual arts, expressive arts, cultural participation, and social prescriptions may benefit older adults’ psychological well-being through emotion regulation, self-expression, cognitive stimulation, skill acquisition, social interaction, and meaning-making (14–29). Recent public health studies similarly indicate that structured arts activities can support health promotion and community engagement among older populations (16–19). Research on heritage arts, intergenerational art engagement, and participatory arts further suggests that the public-health value of arts activities depends not only on aesthetic engagement but also on participatory, achievable, and communicative experiences with social meaning (15, 26–29). However, extant evidence predominantly focuses on generic arts programs, museum-based interventions, integrated arts prescriptions, painting, and expressive arts initiatives; comparatively few studies have directly examined whether ethnic heritage crafts, when culturally tailored, effectively promote health within their communities of origin. Consequently, integrating arts and craft activities, collective engagement, and identity-based meaning into implementable, evaluable community interventions remains a significant gap in healthy aging and culturally adapted public health research.

Research on arts-based public health interventions for older adults spans three closely related areas. First, studies of group arts, visual arts, expressive arts, and arts prescription indicate that structured creative activities may support emotion regulation, self-expression, cognitive activation, social interaction, and meaning-making among older adults (14, 16–25). Second, social prescription and participatory arts studies show that sustained engagement, social capital, and community participation are important conditions through which arts activities may translate into psychosocial benefit (18, 26–29). Third, heritage-oriented arts research shows that cultural and heritage content can be incorporated into evaluable community programs outside conventional clinical settings; for example, Project ARTISAN used an intergenerational arts-and-heritage waitlist-controlled trial in Singapore (15). However, these three strands have mainly examined broad arts programs, intergenerational or museum-based heritage activities, and expressive arts interventions. Less is known about whether a single ethnic heritage craft can be translated into a structured community health intervention in its community of origin. This gap is the focus of the present study, which evaluates Zhuang embroidery, a local ethnic heritage craft, as an integrated program combining craft practice, cultural storytelling, and group exchange among Zhuang adults in Guangxi.

Cultural adaptation frameworks offer a theoretical and practical bridge to this gap. Importantly, cultural adaptation is not merely the superficial incorporation of ethnic symbols or local aesthetics into intervention programs; rather, it requires meaningful integration of intervention materials, activity processes, cultural narratives, and participants’ lived identity experiences. Research on cultural identity and ethnic identification reveals robust associations between cultural identity and well-being, though existing evidence largely remains at the level of mechanistic hypotheses without establishing direct causal pathways (30, 31). In recent years, scholarly attention has turned toward the relationship between intangible cultural heritage and psychosocial well-being; traditional arts, local celebrations, and heritage experiences are increasingly recognized as potential public health resources that sustain cultural continuity, foster social participation, and provide meaning-making opportunities (32, 33). Within the context of China’s intangible cultural heritage, Zhuang embroidery and brocade are distinguished by distinctive patterns, vibrant colors, embedded narratives, and deep-rooted connections to local lived experience; while existing research has concentrated primarily on pattern design, digital dissemination, cultural innovation, and identity construction, these works collectively demonstrate that Zhuang traditional crafts serve as cultural carriers (34–39). In the local Zhuang community context, Zhuang embroidery is a recognizable heritage craft characterized by traditional patterns, color use, and ethnic identity associations (34–39). From a psychological perspective, the retirement transition is associated with changes in work roles, daily structure, social interaction, and purpose in life, which are closely related to dimensions of psychological well-being in later life (1–7). This setting therefore provides an appropriate context for evaluating whether a culturally tailored Zhuang embroidery intervention is feasible and whether it shows preliminary signals for psychological well-being and related psychosocial outcomes.

Against this backdrop, the present study evaluated an eight-week culturally tailored Zhuang embroidery intervention among community-dwelling Zhuang adults in the retirement transition in Guangxi. The program combined embroidery practice, cultural storytelling, and group dialogue and was assessed using a waitlist-controlled quasi-experimental design. The primary aim was to examine preliminary change in psychological well-being, with loneliness, social connectedness, general well-being, ethnic identity, process perceptions, and interview findings treated as secondary or exploratory outcomes. In relation to previous arts-intervention studies, the study’s distinctive contribution lies in evaluating an integrated ethnic heritage-craft program in its local community context while keeping the evidence at the level of feasibility and preliminary psychosocial signals. Accordingly, Hypothesis 1 expected a stronger immediate psychological well-being signal in the intervention group; Hypothesis 2 expected favorable but preliminary changes in secondary psychosocial outcomes; and Hypothesis 3 treated identity-related change as exploratory rather than proof of mediation.

## 2 Materials and methods

### 2.1 Study design

We employed a non-randomized, two-arm, waitlist-controlled quasi-experimental design to evaluate the preliminary effects of the integrated embroidery-based program among community-dwelling Zhuang adults navigating retirement transition in Guangxi. Eighty participants were planned in total, 40 per arm. Both groups underwent assessment at three points: baseline (T0), the end of the eight-week program (T1), and 12 weeks after completion (T2). Once T2 follow-up was done, waitlist participants received the identical program. Reporting drew on guidelines for non-randomized public health interventions and flow diagram conventions, and we deliberately avoided framing the study as a randomized controlled trial (40, 41).

To limit contamination among participants living in the same community, allocation occurred at the community or cluster level rather than individually. The study ran across four de-identified clusters of 20 participants each; C1 and C2 received the intervention, while C3 and C4 formed the waitlist control. No actual community or institutional names appear in this manuscript, with the correspondence kept only in administrative records. This arrangement eased implementation and met ethical obligations to the waitlist, though it also means observed group differences could partly reflect community context and baseline characteristics. We therefore describe the study explicitly as a waitlist-controlled quasi-experiment, not a randomized trial. Primary analyses centered on group, time, and the group-by-time interaction, focusing on how the intervention arm changed relative to controls in psychological well-being and other psychosocial outcomes.

The sample size served preliminary effect estimation and feasibility judgment, not confirmatory efficacy testing. Under an approximate power calculation for two independent groups with two-sided alpha = 0.05, 40 participants per arm would detect a between-group difference of Cohen’s d of roughly 0.63 to 0.65 at 80% power; were the true effect *d* = 0.50, power would drop to roughly 60%. Allowing for about 7.5% attrition at T2, the remaining 37 per arm could still only capture moderate-to-large effects. We therefore set roughly 40 per arm to gauge the direction and magnitude of change in the primary outcome, acknowledging this was insufficient for smaller effects, subgroup contrasts, or exploratory mechanisms such as ethnic identity. All identity, heterogeneity, and process analyses were pre-specified as exploratory, with effect sizes interpreted alongside *p* values and 95% confidence intervals (42). The exploratory heterogeneity screen is reported in Supplementary Table S5.

Ethical review was completed before implementation in line with institutional and community research requirements, and every participant provided written informed consent prior to baseline. Approval details are as follows: ethics committee name, School of Fine Arts and Design, Yulin Normal University; approval or reference number, YLNU-IRB-2025-026; approval date, 18 March 2025. The study constitutes a community public health quasi-experiment rather than a clinical medical trial.

### 2.2 Participants and setting

Participants were permanent residents drawn from four de-identified community clusters in Guangxi. The clusters were community-based recruitment units organized through local community workers and activity sites; actual prefecture/county, street or township, and institutional names were withheld from the public manuscript, with the linkage retained only in administrative files. For each cluster, the administrative record documented the community type, recruitment channel, and basic screening frame used by the local organizers. The target population comprised Zhuang adults aged 55 to 65 navigating retirement transition. Throughout this paper, Zhuang ethnicity functions as a variable tied to cultural adaptation and ethnic identity, not as a marker of vulnerability. All participants needed the capacity to understand study information independently, complete questionnaires, and sign informed consent.

Eligible participants (1) were aged 55 to 65 years; (2) self-identified as Zhuang; (3) had retired or expected to do so within 1 year; (4) passed cognitive screening at the pre-specified threshold; (5) could read the questionnaires or grasp them with researcher assistance; and (6) agreed to take part and signed written consent. Cognitive screening relied on the Mini-Mental State Examination (MMSE) with education-adjusted cutoffs: scores of ≥17 for illiterate participants, ≥20 for primary schooling, and ≥24 for junior secondary education or above indicated a pass (43).

We excluded individuals with (1) severe psychiatric, neurological, or cognitive conditions judged likely to hinder participation or questionnaire completion; (2) ongoing systematic psychotherapy or other interventions that might substantially affect psychological outcomes; (3) serious visual, hearing, or hand-function impairments precluding basic questionnaires or embroidery activities; (4) inability to attend T0, T1, or T2 assessments; or (5) withdrawal of consent during the study. These criteria were necessary for safe participation in an embroidery-based program, but they also mean the sample represents relatively high-functioning and willing community members rather than all retirement-transition adults.

Recruitment was carried out jointly by community workers and the research team through community notice, on-site explanation, and referral within the eligible population frame. Potential participants learned about study aims, intervention content, assessment schedules, possible benefits and risks, confidentiality safeguards, and their right to withdraw at any point. Because participation required willingness to attend repeated sessions and complete questionnaires, volunteer and activity-preference bias could not be excluded. Pilot participants stayed out of the formal N = 80 analysis; piloting served only to check questionnaire comprehension, administration time, missingness, and ceiling or floor risks.

### 2.3 Intervention

The intervention arm received the eight-week culturally tailored Zhuang embroidery program. Sessions ran once weekly for roughly two hours each, held in community activity rooms or cultural centers, with embroidery serving as the activity vehicle. Delivery involved trained researchers working alongside community embroiderers or instructors versed in both craft technique and cultural narration. Every session revolved around three interlocking components: hands-on embroidery, cultural storytelling, and group exchange.

The hands-on component emphasized achievable, low-threshold embroidery tasks progressing in stages. Patterns and materials were matched to participants’ age, eyesight, fine motor ability, and prior craft experience, deliberately steering clear of anything resembling a high-skill competition. For cultural storytelling, instructors offered brief talks on pattern meanings, traditional tales, Zhuang life memories, and local symbolism, helping participants see how the activity connected with their own cultural experience. Group exchange, in turn, invited people to share what they had learned, cultural recollections, feelings about finished pieces, and everyday changes noticed since joining.

By design, the program integrated these three components rather than pitting them against one another. Because making, storytelling, and social exchange occurred together, the waitlist-controlled design could not disentangle the separate contributions of cultural content, generic handcraft, and group socializing. All effect interpretations in this paper are therefore confined to the preliminary impact of the integrated intervention as a whole.

Fidelity was monitored through session scripts, attendance logs, completion records, documentation of cultural content delivery, and notes on protocol deviations. For each participant, researchers tracked attendance across the eight sessions, completion status, timing and reasons for any dropout, and deviations from protocol. Completing the intervention was operationally defined as attending at least six sessions. Waitlist participants kept up their ordinary routines and community activities during the study period, receiving no embroidery program from us until T2 follow-up concluded, after which they were offered the identical arrangement. Ordinary community activities were not prohibited or systematically standardized, so usual-activity variation in the waitlist arm was treated as part of the pragmatic community context rather than an active control condition. A de-identified session framework is provided in Supplementary File 1 (see Table 1).

**Table 1:** Culturally tailored Zhuang embroidery intervention framework.

Component	Main content	Delivery format	Records and quality control
Hands-on embroidery	Learning basic stitches; completing simplified patterns or staged pieces	Weekly demonstration by embroiderers or instructors with in-session practice	Attendance, completion, difficulties, and protocol deviations recorded
Cultural storytelling	Pattern meanings, traditional tales, local life memories, and cultural symbols	Brief talks embedded in each session, tied to that week’s pattern or piece	Whether storytelling followed the script recorded
Group exchange	Sharing making experiences, cultural memories, meaning of works, and life reflections	Structured discussion or free conversation within sessions	Participation and representative feedback recorded
Process manipulation check	Whether participants perceived the storytelling, embroidery, and exchange components	Completed by the intervention group at T1	Used to interpret intelligibility, not as an efficacy outcome
Waitlist arrangement	Ordinary daily and community life during the study; identical program after T2	Delayed intervention	Ethical balance, not an active control

The table summarizes the integrated intervention components and implementation records; components were delivered together and were not evaluated separately.

### 2.4 Measures and study procedures

All participants completed primary and secondary outcome measures at T0, T1, and T2. Standardized scales were administered in validated or licensed Chinese versions, with item-level raw scores retained so that totals, subscale scores, and Cronbach’s alpha could later be computed.

The primary outcome, psychological well-being, was measured with the Ryff Scales of Psychological Well-Being. The instrument spans autonomy, environmental mastery, personal growth, positive relations with others, purpose in life, and self-acceptance, higher scores reflecting greater well-being (1). We adopted the 18-item Chinese version, computing the total and six dimension scores per the manual, with reverse-keyed items converted under Chinese scoring rules.

Secondary outcomes covered general mental well-being, loneliness, and social connectedness. General well-being was assessed with the Chinese WEMWBS and WHO-5; higher WEMWBS scores indicate better mental well-being, and WHO-5 scores were summed as a 0 to 25 total score, with higher scores indicating better general well-being (44, 45). Loneliness was captured by the Chinese ULS-8, on which higher scores mean greater loneliness, so a decline represents improvement (46). The Chinese Social Connectedness Scale measured connectedness, higher scores signaling stronger ties (47). Since healthy community samples may start out relatively happy, we planned to report ceiling effects for WEMWBS, WHO-5, and related outcomes; for the ULS-8, attention turned instead to floor effects and possible underestimation from social desirability.

Ethnic identity served as the exploratory mechanism variable, measured with the Chinese Multigroup Ethnic Identity Measure (MEIM) or a domestically validated equivalent, higher scores indicating stronger identity. Its role was to probe whether identity change tracked well-being gains, not to establish causal mediation. The manipulation check, completed by the intervention arm at T1, asked whether participants clearly noticed the cultural storytelling; how helpful they found the embroidery, storytelling, and group exchange respectively; whether the experience would have felt different had it been plain handcraft without Zhuang stories; and whether the activity deepened their closeness to or identification with Zhuang culture. These items were researcher-devised process indicators, not an efficacy scale.

Internal consistency was assessed at each time point. Cronbach’s alpha ranged from 0.89 to 0.93 for Ryff psychological well-being, 0.84 to 0.88 for mental well-being measured by the WEMWBS, 0.69 to 0.74 for loneliness measured by the ULS-8, 0.76 to 0.79 for social connectedness, 0.63 to 0.77 for general well-being measured by the WHO-5, and 0.83 to 0.85 for ethnic identity. Time-specific Cronbach’s alpha values, item-total correlations, and floor and ceiling diagnostics are reported in Supplementary Table S3.

Qualitative interviews took place at T1 with a purposive subsample of the intervention arm, following a semi-structured guide. The sampling matrix considered sex, age, retirement status, prior embroidery experience, and attendance, aiming to capture varied participation experiences rather than statistical representativeness or saturation. Topics ranged across overall impressions of the 8 weeks, shifts in mood and daily life, cultural memories evoked by embroidery, feelings toward Zhuang culture, the group exchange experience, the sense of completing a piece, purpose in life, and the relative meaning of the three components. Interviews proceeded only after recording consent was obtained, and transcripts were de-identified. Qualitative material served to enrich interpretation of the quantitative findings, never to replace or shore up null results. The semi-structured interview guide is provided in Supplementary File 2.

Procedures unfolded as follows. At T0, participants gave consent and underwent eligibility screening, demographic and background data collection, cognitive screening, and the full battery of scales. T1, at the close of week eight, repeated all scales and added the manipulation check plus subsample interviews for the intervention arm. T2 administered the full battery once more, twelve weeks after the program ended. Administrative records held anonymized IDs, cluster codes, allocation, testing dates, assessor codes, and consent documentation. The key linking real identities to data was stored apart from analytic files, which carried de-identified codes only; electronic files remained accessible to designated team members alone, backed up and version-tracked per data management requirements.

Before analysis, questionnaires were screened using predefined data-quality rules: missing essential identifiers, missing group or time-point information, impossible or out-of-range item responses, excessive item missingness preventing total-score calculation, and patterned responses such as straight-lining were flagged for review. Cases were excluded only when the review indicated that the questionnaire could not support valid scoring or linkage across assessment points. Main outcome analyses used computable scale totals; reliability and floor and ceiling diagnostics used complete item-level records for the relevant scale and time point, so diagnostic sample sizes could be smaller than the number of participants who completed a follow-up questionnaire.

### 2.5 Statistical analysis

Analysis opened with descriptions of participant flow, adherence, attrition, and baseline characteristics. Continuous variables appear as means with standard deviations or medians with interquartile ranges; categorical variables as counts and percentages. Baseline comparisons between arms used *t* tests, Mann–Whitney *U* tests, Chi-square tests, or Fisher’s exact test, depending on variable type. Pre-specified individual-level covariates for adjusted models comprised age, sex, education, living alone, income, and chronic disease status, with age entering as a continuous term and education and income entering as categorical terms so that all reported categories, including non-disclosure of income, remained in the model. Baseline outcome levels were handled through the repeated-measures structure spanning T0, T1, and T2, rather than added again as a separate covariate in the same model. No baseline outcome score and no covariate-by-time interaction terms were included in the primary adjusted models. Imbalanced baseline variables were interpreted alongside Table 2 and model output. Prior embroidery experience was not included in the pre-specified main model because it was not a stratification or confirmatory adjustment factor, but its baseline imbalance was reported and treated as a residual confounding and engagement-related limitation.

**Table 2:** Baseline characteristics of the two groups.

Variable	Intervention (*n* = 40)	Waitlist control (*n* = 40)	*p*-value	Standardized difference
Age, years	60.60 (2.92)	59.17 (3.26)	0.043	0.46
Cognitive screening score	27.88 (1.60)	27.52 (1.88)	0.373	0.20
Ryff psychological well-being total	56.80 (19.34)	63.17 (20.61)	0.158	−0.32
WEMWBS well-being total	35.80 (9.14)	39.45 (11.71)	0.125	−0.35
ULS-8 loneliness total	19.32 (4.98)	18.00 (4.25)	0.205	0.29
Social connectedness total	25.15 (8.55)	25.77 (7.56)	0.730	−0.08
WHO-5 well-being total	11.55 (6.42)	12.12 (5.60)	0.671	−0.10
Ethnic identity total	35.77 (10.78)	35.52 (9.34)	0.912	0.02
Female	23 (57.5%)	27 (67.5%)	0.488	—
Living alone	9 (22.5%)	8 (20.0%)	1.000	—
Chronic disease	15 (37.5%)	14 (35.0%)	1.000	—
Prior embroidery experience	Occasionally 21 (52.5%); never 15 (37.5%); often 4 (10.0%)	Never 21 (52.5%); occasionally 10 (25.0%); often 9 (22.5%)	0.033	—
Education	Middle school 17 (42.5%); high/vocational 14 (35.0%); college or above 6 (15.0%); primary or less 3 (7.5%)	High/vocational 14 (35.0%); middle school 12 (30.0%); college or above 8 (20.0%); primary or less 6 (15.0%)	0.542	—
Income	3,000–5,999: 15 (37.5%); 6,000–8,999: 13 (32.5%); below 3,000: 7 (17.5%); prefer not: 3 (7.5%); 9,000+: 2 (5.0%)	3,000–5,999: 15 (37.5%); 6,000–8,999: 11 (27.5%); below 3,000: 6 (15.0%); prefer not: 5 (12.5%); 9,000+: 3 (7.5%)	0.918	—

Continuous variables are shown as mean (SD); categorical variables as *n* (%).

Primary analyses relied on linear mixed models to draw fully on the repeated measures at T0, T1, and T2 (48). Fixed effects covered group, time, their interaction, and the pre-specified individual covariates, while a random intercept for each participant accommodated the repeated-measures structure. The adjusted estimates in Table 3 are model-based group-by-time interaction contrasts conditional on the listed covariates and available repeated observations. Because T1 had complete scale totals in both arms, the corrected adjusted T1 point estimates coincide with the unadjusted change-score contrasts; T2 estimates may differ slightly because the mixed model uses all available repeated observations rather than only paired T2 completers. The core model took the form:

$$\begin{array}{l} \text{outcome}=\text{group}+\text{time}+\text{group}\times \text{time}\\ +\text{covariates}+\text{random intercept}(\mathrm{id}) \end{array}$$

**Table 3:** Between-group differences in change and adjusted model results for primary and secondary outcomes.

Outcome	Contrast	Intervention change, mean (SD)	Waitlist change, mean (SD)	Between-group difference (95% CI)	d	Unadjusted p	Adjusted model estimate (95% CI)	Adjusted p	Analysis n (I/W)
Ryff well-being	T1-T0	14.29 (14.02)	2.10 (17.13)	12.18 (5.22 to 19.15)	0.78	<0.001	12.18 (6.28 to 18.09)	<0.001	40/40
Ryff well-being	T2-T0	8.85 (13.75)	2.18 (11.49)	6.68 (0.80 to 12.55)	0.53	0.027	6.22 (0.14 to 12.29)	0.045	37/37
WEMWBS	T1-T0	7.48 (5.63)	3.54 (8.32)	3.94 (0.78 to 7.10)	0.55	0.016	3.94 (0.62 to 7.25)	0.020	40/40
WEMWBS	T2-T0	4.70 (8.24)	3.77 (7.85)	0.94 (−2.79 to 4.66)	0.12	0.619	0.90 (−2.51 to 4.31)	0.605	37/37
ULS-8 loneliness (OLS fallback)	T1-T0	−1.65 (5.10)	0.88 (5.28)	−2.53 (−4.84 to −0.22)	−0.49	0.032	−2.53 (−5.23 to 0.17)	0.067	40/40
ULS-8 loneliness (OLS fallback)	T2-T0	1.26 (5.20)	2.08 (5.52)	−0.82 (−3.30 to 1.67)	−0.15	0.513	−0.61 (−1.27 to 0.05)	0.072	37/37
Social connectedness	T1-T0	6.01 (8.10)	3.35 (5.54)	2.66 (−0.43 to 5.75)	0.38	0.091	2.66 (−0.11 to 5.43)	0.060	40/40
Social connectedness	T2-T0	2.84 (6.34)	1.00 (5.75)	1.84 (−0.96 to 4.65)	0.30	0.195	1.67 (−1.17 to 4.52)	0.250	37/37
WHO-5	T1-T0	0.61 (4.13)	1.19 (4.92)	−0.59 (−2.61 to 1.43)	−0.13	0.565	−0.59 (−2.62 to 1.44)	0.570	40/40
WHO-5	T2-T0	1.80 (5.63)	0.16 (4.93)	1.64 (−0.82 to 4.09)	0.31	0.188	1.69 (−0.39 to 3.78)	0.111	37/37
Ethnic identity	T1-T0	5.27 (7.64)	1.70 (5.85)	3.57 (0.54 to 6.60)	0.52	0.022	3.57 (0.68 to 6.46)	0.015	40/40
Ethnic identity	T2-T0	2.62 (6.61)	0.36 (7.03)	2.26 (−0.91 to 5.42)	0.33	0.159	2.03 (−0.94 to 4.99)	0.181	37/37

Unadjusted contrasts are between-group differences in change scores. Adjusted estimates are group-by-time interaction contrasts from repeated-measures models adjusted for age, sex, education, living alone, income, and chronic disease. T1 analyses included 40 participants per group; T2 analyses included 37 participants per group unless otherwise indicated. ULS-8 adjusted estimates used covariate-adjusted OLS with cluster-robust standard errors because the mixed model returned a singular fit. Because only four clusters were available, these cluster-robust standard errors should be interpreted cautiously. Lower ULS-8 scores indicate less loneliness. Additional Ryff sensitivity checks, including complete-case, per-protocol, and baseline-adjusted ANCOVA analyses, are summarized in Supplementary Table S8.

Interest centered on the group-by-time interaction, which gauges how the intervention arm changed over time relative to controls. The Ryff total served as the primary outcome; key secondary outcomes were the WEMWBS, ULS-8, social connectedness, and WHO-5. Models reported immediate (T1) and sustained (T2) effects, giving adjusted between-group differences, 95% confidence intervals, *p* values, and Cohen’s d. For the ULS-8, we made explicit that higher scores mean greater loneliness, so negative change signals improvement.

Because allocation rested on four clusters nested within group, the sample could not support stable estimation of cluster-level random effects. The main model therefore kept individual random intercepts as the repeated-measures structure, with cluster ID serving chiefly to document allocation and potential community-level confounding; this constraint is taken up in the Discussion as a key limitation of non-randomized cluster assignment (49). Missing data handling leaned on the mixed model’s capacity to use all available repeated measures under a missing-at-random assumption conditional on observed information; we did not assume that missingness was formally missing completely at random. Given the small number of T2 non-completers, no outcome imputation or multiple-imputation sensitivity analysis was performed.

Three sensitivity analyses were used overall: a complete-case analysis restricted to those with valid primary outcome totals at all three points, a per-protocol analysis defining intervention completers as those who attended at least six sessions and finished T1 compared against the waitlist arm, and the baseline-adjusted Ryff ANCOVA models described below.

Sensitivity analyses used baseline-adjusted ANCOVA models for Ryff psychological well-being at T1 and T2. Each model included the post-baseline Ryff total score as the outcome, group as the main factor, and baseline Ryff score as a covariate. Fully adjusted models additionally included age, sex, education, living alone, income, and chronic disease. Because prior embroidery experience differed between groups at baseline, these models were repeated with prior embroidery experience added as an additional covariate. The analyses assessed the robustness of the primary repeated-measures findings.

Identity mechanism analyses were pre-specified as exploratory. Priority went to the correlation between change in ethnic identity and change in Ryff scores; where data quality and sample size permitted, an exploratory mediation model could further examine relations among group, identity change, and well-being change. All such analyses kept to the language of correlation, possible mechanism, and exploration, never claiming causal mediation for ethnic identity. Manipulation check items were summarized descriptively, with their association with identity change left open to exploration.

Interview material underwent thematic analysis (50). De-identified transcripts were read repeatedly, initial codes were generated from meaning units, related codes were organized into candidate themes, and theme names were refined against the interview guide and the quantitative results. Coding decisions and exemplar excerpts were retained as an audit trail. Qualitative findings appear as themes with brief quotations, each attributed to a pseudonym or code so that no identifying detail surfaces.

All tests were two-sided with significance set at alpha = 0.05. Given the preliminary nature of the study, effect sizes and 95% confidence intervals were interpreted alongside *p*-values rather than in isolation. No Bonferroni or false-discovery-rate correction was applied across the secondary and exploratory outcomes; these results were therefore read as hypothesis-generating rather than confirmatory. Analyses were run in IBM SPSS Statistics 29.0.

## 3 Results

Results are organized by participant flow and baseline characteristics, outcomes and sensitivity analyses, identity and process findings, and qualitative interview findings. Because allocation occurred at the community-cluster level across four clusters, between-group differences should be interpreted as preliminary signals that may reflect both intervention exposure and community-level differences.

### 3.1 Participant flow, adherence, and baseline characteristics

Eighty-eight questionnaires came back initially; eight fell out at quality screening owing to incomplete information, missing essential identifiers or assessment timing, out-of-range responses, or patterned response behavior requiring exclusion, leaving a final analytic sample of 80, split evenly between the intervention arm (*n* = 40) and waitlist control (*n* = 40). Every participant in both arms completed the immediate post-intervention assessment (T1), a 100.0% completion rate. At follow-up (T2), 37 per arm finished the questionnaires, retention standing at 92.5% in each group. The six T2 non-completions were attributed to loss to contact (*n* = 3), scheduling conflicts (*n* = 2), and health reasons (*n* = 1). Figure 1 charts participant flow. For scale-level reliability and floor and ceiling diagnostics, the T2 denominator was the complete item-level diagnostic subset (*n* = 67), whereas 74 participants completed follow-up questionnaires and had computable scale totals for the main outcome analyses. Accordingly, the analytic denominators were *n* = 80 at baseline, *n* = 74 for computable T2 scale totals in the main outcome analyses, *n* = 67 for complete-item T2 reliability and floor and ceiling diagnostics, and 37 per arm for T2 follow-up completers (Supplementary Table S4).

**Figure 1:**
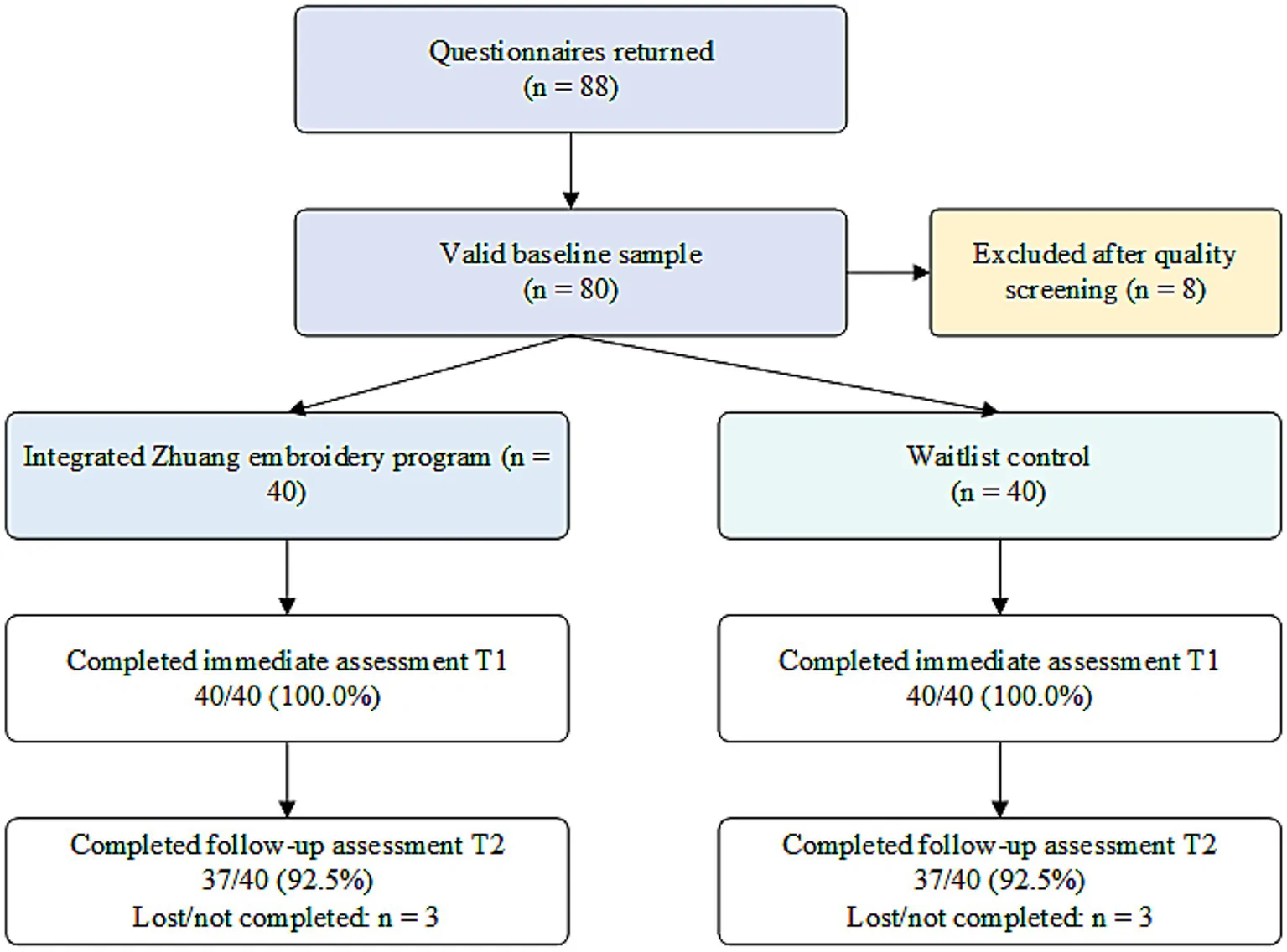
Participant enrollment, allocation, and follow-up flow diagram.

At baseline, the arms were broadly comparable on sex, education, income, living alone, chronic disease, cognitive screening, psychological well-being, subjective well-being, loneliness, social connectedness, general well-being, and ethnic identity (Table 2), but several imbalances deserve mention. The intervention group was slightly older than the waitlist group (60.60 (2.92) vs. 59.17 (3.26) years; *p* = 0.043; SMD = 0.46). Prior embroidery experience was unevenly distributed between groups (*p* = 0.033): among intervention participants, 15 (37.5%) had never embroidered, 21 (52.5%) did so occasionally, and 4 (10.0%) often, whereas waitlist figures were 21 (52.5%), 10 (25.0%), and 9 (22.5%). The intervention arm also had a lower baseline psychological well-being score (56.80 vs. 63.17; *p* = 0.158; SMD = −0.32), a non-significant but practically relevant difference that raises a regression-to-the-mean concern. These imbalances are considered in the adjusted analyses and in the interpretation of residual confounding.

### 3.2 Primary and secondary outcomes with sensitivity analyses

Ryff psychological well-being, the primary outcome, showed the clearest between-group difference at T1. Mean T1-T0 change was 14.29 (14.02) points in the intervention arm and 2.10 (17.13) in the waitlist arm, giving a between-group difference of 12.18 points (95% CI: 5.22 to 19.15; Cohen’s *d* = 0.78; *p* < 0.001). In the adjusted repeated-measures mixed model, which included age, sex, education, living alone, income, and chronic disease, the T1 group-by-time interaction was similar (estimate = 12.18, 95% CI: 6.28 to 18.09, *p* < 0.001). At T2, the between-group difference was smaller: the T2-T0 change contrast was 6.68 points (95% CI: 0.80 to 12.55; *d* = 0.53; *p* = 0.027), and the adjusted interaction estimate was 6.22 (95% CI: 0.14 to 12.29, *p* = 0.045). The primary repeated-measures model therefore indicated a clear immediate Ryff signal and a weaker follow-up signal.

Secondary outcomes diverged more sharply. WEMWBS showed a T1 advantage: mean change ran 7.48 (5.63) points in the intervention arm versus 3.54 (8.32) in controls, a difference of 3.94 points (95% CI: 0.78 to 7.10, *d* = 0.55, *p* = 0.016), with the adjusted interaction likewise significant (estimate = 3.94, 95% CI: 0.62 to 7.25, *p* = 0.020). By T2, however, that gap had narrowed markedly and lost significance (difference = 0.94, 95% CI: −2.79 to 4.66, *p* = 0.619; adjusted estimate = 0.90, 95% CI: −2.51 to 4.31, *p* = 0.605), and the intervention group showed greater dispersion in T2 change than at T1, suggesting more heterogeneous follow-up responses. For loneliness (ULS-8), unadjusted T1 comparison showed a steeper decline among intervention participants (−2.53 points, 95% CI: −4.84 to −0.22, *d* = −0.49, *p* = 0.032); in the fallback adjusted OLS model, though, the interaction only approached significance (estimate = −2.53, 95% CI: −5.23 to 0.17, *p* = 0.067), and the T2 difference had faded (adjusted estimate = −0.61, 95% CI: −1.27 to 0.05, *p* = 0.072). Social connectedness moved favorably at both points yet did not cross conventional thresholds (adjusted T1 *p* = 0.060; adjusted T2 *p* = 0.250). WHO-5 revealed no clear advantage at either point; scale diagnostics moreover put its Cronbach’s alpha at 0.63 at T1, counseling caution in reading those particular results.

Overall, the pattern was selective rather than broadly consistent across psychosocial outcomes. Psychological well-being showed the most consistent immediate signal; WEMWBS and loneliness showed short-term favorable movement only, whereas evidence for social connectedness and WHO-5 remained weak and most secondary signals declined by follow-up. Table 3 presents the main results, and Figure 2 shows the trajectories. Complete-case and per-protocol sensitivity analyses were directionally consistent with the primary repeated-measures model; these estimates are summarized alongside the baseline-adjusted ANCOVA models in Supplementary Table S8. The baseline-adjusted ANCOVA sensitivity analysis below further qualified the follow-up result.

**Figure 2:**
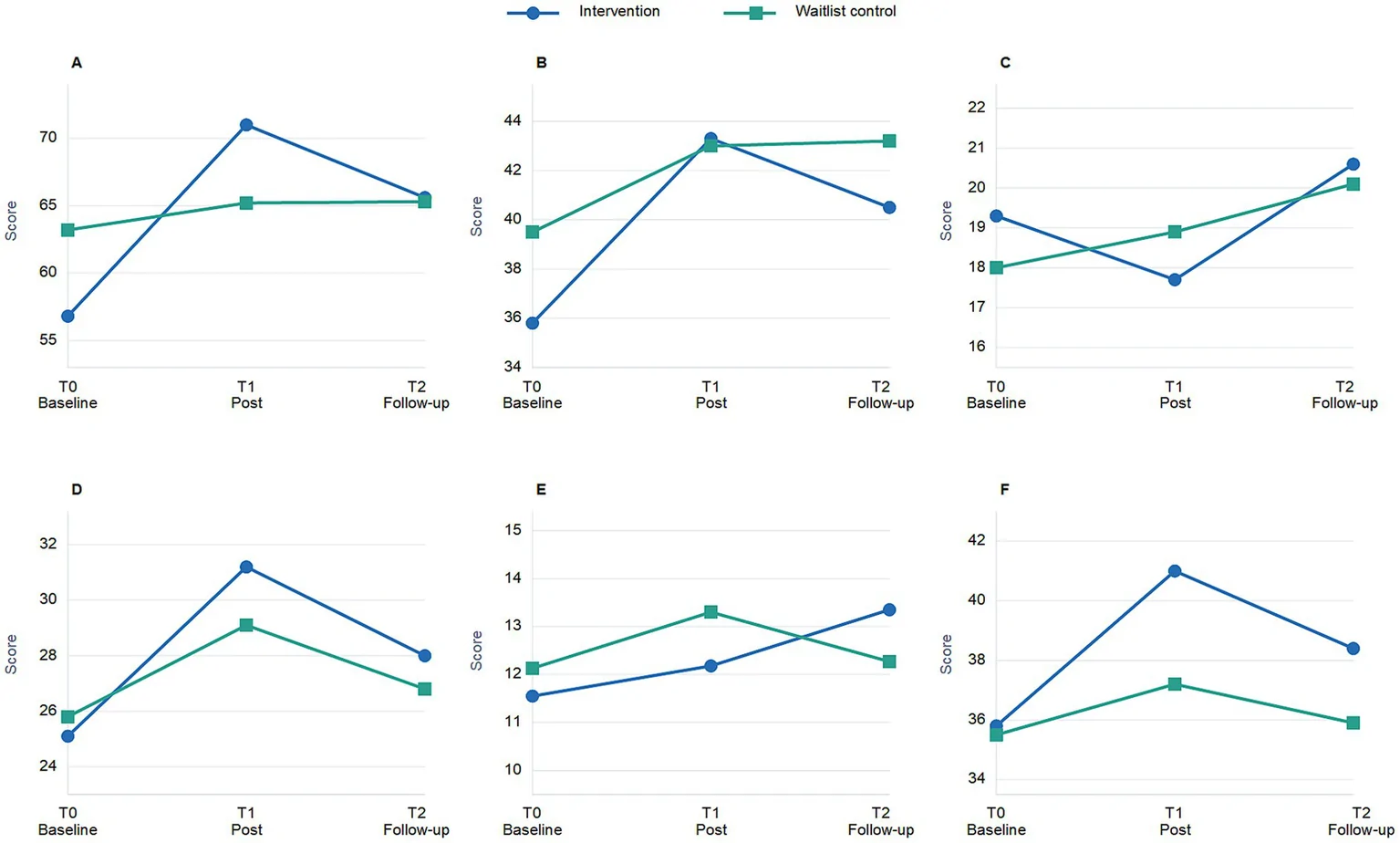
Descriptive mean trajectories of primary and secondary outcomes at T0, T1, and T2 in the two groups. Error bars are not displayed to preserve readability; sample sizes, inferential contrasts, and 95% confidence intervals are reported in Table 3. **(A)** Ryff psychological well-being. **(B)** WEMWBS well-being. **(C)** ULS-8 loneliness. **(D)** Social connectedness. **(E)** WHO-5 well-being. **(F)** Ethnic identity.

In the baseline-adjusted ANCOVA sensitivity analyses, T1 estimates were similar across the baseline-only model (adjusted mean difference = 9.63, 95% CI: 3.52 to 15.73, *p* = 0.002; *n* = 80), the fully adjusted model (adjusted mean difference = 9.86, 95% CI: 3.69 to 16.04, *p* = 0.002; *n* = 80), and the model additionally adjusted for prior embroidery experience (adjusted mean difference = 10.25, 95% CI: 3.79 to 16.72, *p* = 0.002; *n* = 80). T2 estimates were smaller and not statistically significant in the baseline-only model (adjusted mean difference = 4.12, 95% CI: −1.18 to 9.41, *p* = 0.125; *n* = 74), the fully adjusted model (adjusted mean difference = 4.43, 95% CI: −1.36 to 10.23, *p* = 0.131; *n* = 74), and the model additionally adjusted for prior embroidery experience (adjusted mean difference = 4.33, 95% CI: −1.74 to 10.39, *p* = 0.159; *n* = 74). Full sensitivity results are shown in Supplementary Table S8.

### 3.3 Ethnic identity and process evaluation

Ethnic identity, the exploratory outcome, showed short-term positive movement. At T1, intervention scores rose 5.27 (7.64) points from baseline against 1.70 (5.85) among controls, a difference of 3.57 points (95% CI: 0.54 to 6.60, *d* = 0.52, *p* = 0.022). The adjusted mixed model gave the same T1 point estimate and remained significant (estimate = 3.57, 95% CI: 0.68 to 6.46, *p* = 0.015). At T2, however, neither unadjusted nor adjusted analyses showed a clear sustained effect. The identity hypothesis therefore receives only a short-term exploratory signal: the data do not establish that identity change was durable or that it drove the well-being effect.

Process evaluation painted a broadly positive picture of how participants perceived the storytelling, needlework, and group interaction. Clarity of cultural content averaged 4.33 (0.53); rated importance came to 4.25 (0.71) for needlework, 4.33 (0.57) for cultural content, and 3.68 (0.94) for group interaction, with an overall process score of 4.12 (0.36). These high ratings suggest good perceived acceptability, but they also raise the possibility of ceiling effects, courtesy bias, or social desirability in intervention-arm-only process ratings.

These findings suggest that the integrated Zhuang embroidery activity, as a minority-arts and local heritage-craft vehicle, was generally comprehensible and acceptable to participating older community members. The exploratory correlations told a more muted story. Across the full sample, T1 identity change correlated weakly and non-significantly with Ryff change (*r* = 0.21, *p* = 0.064, *n* = 80); within the intervention arm alone the link was weaker still (*r* = 0.16, *p* = 0.331, *n* = 40). Nor did the overall process score relate to identity change (*r* = 0.19, *p* = 0.238, *n* = 40). Identity results therefore fail to support the pre-specified exploratory mechanism and can only seed future hypotheses. The safer conclusion is that the activity carried cultural meaning and community acceptability, though the design could not apportion credit among the art’s cultural character, the making process, and group interaction, nor sustain any formal mediation claim.

Full process evaluation results appear in Supplementary Table S1; exploratory correlations between identity change and psychological outcomes in Supplementary Table S2; and the exploratory mechanism path in Supplementary Figure S1.

### 3.4 Qualitative interview findings

To enrich the quantitative picture, ten intervention participants sat for semi-structured interviews at T1. Purposive sampling sought spread across sex, age, retirement status, prior embroidery experience, and attendance; the sample was meant to display varied experiences and acceptability, not to stand for the whole arm. Thematic analysis yielded four themes: cultural continuity and a felt closeness of identity; mastery and daily rhythm gained from finishing pieces; companionship and mutual encouragement through group exchange; and storytelling that set the activity apart from plain handcraft, though experiences varied person to person. Interviewee details and coding appear in Supplementary Tables S6 and S7.

Most interviewees tied the activity to childhood memories, women’s experiences within their families, or the meanings of Zhuang patterns, crediting the storytelling with deepening its significance. As one put it, “Only after the teacher explained what the patterns meant did I feel these things were connected to our own lives” (I01). Another described how finishing pieces restored routine and a sense of accomplishment amid the retirement transition: “Having something waiting for me each week settles my heart” (I06).

Interviews also pointed to group exchange as a likely wellspring of relief from loneliness and of social connection. “Everyone chats while stitching, and time never drags” (I04), one participant observed. Some allowed that ordinary handcraft might bring interest and company too, yet felt the patterns, storytelling, and ethnic memory lent this program a distinctly local cultural meaning; others spoke of frustration with fine stitches, tired eyes, or slow hands. The qualitative material thus supports reading Zhuang embroidery as a culturally meaningful, community-acceptable vehicle for minority-arts intervention. Absent a plain handcraft arm or purely social arm, however, these data cannot apportion the separate contributions of the art’s cultural character, the making itself, and group interaction.

## 4 Discussion

### 4.1 Principal findings and comparison with prior research

This study appraised the preliminary effects of an embroidery-anchored program blending practice, cultural storytelling, and group exchange among Zhuang adults navigating retirement transition in Guangxi communities. The primary result was clearest for psychological well-being: scores on the primary well-being measure improved noticeably by the end of the 8 weeks; the repeated-measures model suggested a weaker follow-up signal, whereas baseline-adjusted sensitivity analysis attenuated that follow-up difference. Secondary outcomes were mixed. Mental well-being improved at program end but not at follow-up; loneliness improved in the unadjusted T1 contrast but only approached significance after adjustment and did not hold at T2; social connectedness moved favorably without crossing conventional thresholds; and general well-being showed no clear advantage. Ethnic identity showed a short-term adjusted signal, but exploratory correlations did not support it as a stable mechanism. The overall pattern therefore supports a selective primary-outcome signal, not broad psychosocial improvement across all outcomes.

This selective pattern is consistent with the literature reviewed in the Introduction. Studies of group arts, arts prescription, and participatory arts suggest that creative activities may support older adults’ mental health through emotion regulation, self-expression, cognitive activation, social interaction, and meaning-making (14–29). The present findings are more modest: a culturally meaningful heritage-craft activity in a local community was associated with short-term gains in developmental psychological well-being. The weaker findings for mental well-being, loneliness, social connectedness, and general well-being are equally informative. These outcomes capture different dimensions of experience: psychological well-being emphasizes purpose, mastery, growth, and positive relations; mental and general well-being reflect broader affective states; loneliness and connectedness depend more directly on sustained social relationships. A brief eight-week craft program may therefore be better positioned to influence mastery, meaning, and purpose than to produce durable change in broader affective well-being or social relationship patterns. This interpretation is consistent with reviews reporting heterogeneous loneliness-intervention effects and the need for sustained social opportunities (8–11, 13).

### 4.2 Possible mechanisms and public health implications

Gains in well-being likely arose from the joint working of craft-making, group interaction, and cultural meaning. Hands-on embroidery brought skill learning, redirected attention, finished pieces, and a sense of mastery; group exchange opened shared learning, peer support, and fresh avenues for community contact; the storytelling, for its part, may have tied the activity to ethnic memory, local lived experience, and identity, strengthening both motivation and felt meaning (29–33). The interview findings support this interpretation at the level of participant experience: interviewees described cultural stories, recognizable patterns, completed pieces, and group conversation as reasons the activity felt meaningful, acceptable, and distinct from ordinary handcraft. For people moving through retirement, such activities may help rebuild daily structure, accomplishment, and purpose once the working role has ended (2–7).

These accounts remain candidate mechanisms, not verified causal pathways. Because the waitlist-controlled quasi-experimental design bundled making, storytelling, and exchange, the relative contribution of each component cannot be separated (40, 41). Process data and interviews support cultural fit and community acceptability, but they do not establish mediation, component-specific effects, or the superiority of embroidery over other forms of craft or social activity (30, 31).

From a public health perspective, the study tested a low-threshold form of community health promotion built on local cultural resources. Retirement-transition adults may benefit from sustainable activities that support psychological resources, daily structure, and social contact (9, 27). The program required modest infrastructure: community activity rooms or cultural centers, local staff, and instructors familiar with the craft. For Zhuang communities in Guangxi, translating local heritage craft into structured health promotion may strengthen cultural acceptability and willingness to participate, although this claim requires confirmation in larger and more rigorous studies (32–39). Given the unstable secondary and follow-up results, the program is best understood as an early retirement-transition support option rather than an established transferable model. Lasting gains in loneliness and connection would likely require maintenance features such as ongoing groups, community exhibitions, peer support, or family involvement (8, 13, 27).

### 4.3 Strengths and limitations

Strengths of this work include its focus on a preventive population, Zhuang adults in retirement transition, and its translation of an ethnic heritage art into a structured community intervention. Assessment at three time points separated immediate from short-term follow-up effects; process evaluation and exploratory identity analyses helped gauge whether participants grasped and accepted the components. Rather than merely describing heritage transmission or cultural experience, the study appraised a culturally tailored intervention against public health outcomes, sketching an initial empirical route for local cultural resources within healthy aging.

Clear limitations remain. First, the non-randomized waitlist design cannot fully rule out selection bias, community-context differences, or unmeasured confounding; with only four de-identified clusters nested within group, cluster-level random effects could not be stably estimated, and even after individual covariate adjustment the causal footing falls short of a randomized trial (40, 41). Community names were withheld for confidentiality, but this also limits readers’ ability to judge the recruitment frame and setting heterogeneity. Second, the sample was small, 40 per arm shrinking to 37 at follow-up, adequate for gauging preliminary direction but not for smaller effects, subgroup contrasts, multiplicity-adjusted secondary testing, or mechanism models (42). Third, drawn from a single region and few communities, and restricted to cognitively intact volunteers without major sensory or hand-function barriers, the findings may not travel to other ethnic areas, urban settings, non-Zhuang populations, or less functional retirement-transition adults. Fourth, baseline age, prior embroidery experience, and baseline psychological well-being scores differed between arms; because embroidery experience was never designed as a confirmatory stratification factor, related heterogeneity findings serve only as exploratory leads, and regression-to-the-mean or engagement-related confounding cannot be ruled out. The T2 psychological well-being difference also did not remain statistically significant in the baseline-adjusted ANCOVA, which is consistent with a possible regression-to-the-mean contribution to the follow-up pattern. Fifth, the waitlist design can neither apportion the contributions of cultural character, making process, and group interaction, nor establish the increment of embroidery over ordinary handcraft; the usual-activity control condition was pragmatic but not a fully characterized active comparator.

Beyond these, all primary and secondary outcomes rested on self-report, leaving them open to social desirability, researcher expectancy, and repeated-testing effects; sensitive outcomes like loneliness may well be understated (44–47). Scale diagnostics flagged low internal consistency for the general well-being measure at T1. The loneliness measure also showed measurement and modeling fragility: the minimum corrected item-total correlation was low at baseline (0.16), and the adjusted mixed model required a supporting OLS approach after mixed-model singularity. These results should therefore be read cautiously and not as confirmatory evidence for loneliness reduction. Missing-data handling relied on observed repeated measures without multiple imputation, and the small number of T2 losses does not by itself prove that missingness was completely random. That several secondary outcomes and the identity analyses fell short of significance points to selective rather than sweeping effects, especially because no multiplicity correction was applied. The interview subsample was small and purposively selected; qualitative material served to illuminate experience and acceptability, and can neither substitute for the quantitative outcomes nor prop up null or unstable results (50).

### 4.4 Directions for future research

Future work should adopt more rigorous multi-center, multi-community randomized or cluster-randomized designs to curb selection bias and community-level confounding (40, 41, 49). More importantly, multi-arm studies could dismantle the intervention into its parts, comparing, say, embroidery plus storytelling plus exchange, plain handcraft plus exchange, social contact alone, and waitlist or usual activity, thereby testing the relative contributions of cultural character, making, and social interaction. Such a design would move toward answering whether embroidery, as a minority-arts vehicle, carries incremental value beyond generic craft and group activity.

Follow-up windows should also be lengthened, with low-intensity maintenance built in after the program ends, works-sharing sessions, community exhibitions, peer support, or family involvement, to test whether gains in well-being, loneliness, and connection endure. Mechanism studies ought to be preregistered, specifying measurement points and analytic paths for identity, connection, engagement, and mastery, with samples sized to sustain mediation or moderation analyses. Finally, cultural adaptation research must resist reducing minority culture to a mere instrument. Embroidery here is better understood as a community resource knitting together local culture, handcraft, group ties, and life meaning; future designs should draw community members, embroiderers, cultural workers, and older participants into co-design, raising cultural respect, acceptability, and sustainability.

## 5 Conclusion

This waitlist-controlled quasi-experiment indicates that the integrated Zhuang embroidery program was feasible among community-dwelling Zhuang adults in retirement transition in Guangxi. Hypothesis 1 received preliminary support for an immediate psychological well-being signal: the intervention group improved more than the waitlist group at T1, and the baseline-adjusted sensitivity analysis supported this immediate difference. The follow-up signal was weaker and was attenuated after baseline adjustment. Hypothesis 2 received only partial and time-limited support: mental well-being and loneliness showed short-term favorable movement, but social connectedness and general well-being were weak, and most secondary effects were not sustained at T2. Hypothesis 3 received limited support only for a short-term ethnic identity increase; exploratory correlations did not establish identity change as a causal driver of well-being gains.

Process evaluation and interviews indicated that participants recognized and accepted the storytelling, needlework, and group interaction, supporting the cultural fit and acceptability of embroidery as a local heritage-craft vehicle for community health promotion. The design, however, could not separate the contributions of cultural character, making, and group interaction, nor could it exclude expectancy effects, community-level differences, residual confounding, or regression-to-the-mean influences. Conclusions should therefore remain confined to feasibility and preliminary effect signals. Larger samples, more communities, multi-arm randomized designs, active comparators, and longer follow-up are needed to confirm stable effects, component contributions, and underlying mechanisms.

## Data Availability

The raw data supporting the conclusions of this article will be made available by the authors, without undue reservation.
