# Supplementary material for: A culturally tailored Zhuang embroidery intervention for psychological well-being among Zhuang adults in the retirement transition in Guangxi: a waitlist-controlled quasi-experimental study
Source: Front Public Health. 2026 Sep 17;14:1945265. doi: 10.3389/fpubh.2026.1945265 (PMC13626903; doi:10.3389/fpubh.2026.1945265)
Supplement: Supplementary file 1 [file Data_Sheet_1.PDF]

Supplementary Material

1 Supplementary Material Overview

This supplement carries the process evaluation, exploratory mechanisms, scale diagnostics, data quality checks, model-audit contrasts, and heterogeneity screening that the main text could not accommodate in full. All supplementary analyses serve to clarify the robustness and boundaries of the main findings; none advances new primary conclusions.

Item	Title	Linked section	Main purpose
Supplementary Table S1	Process evaluation and manipulation check results	Results 3.3	Perceptions of cultural storytelling, needlework, and group interaction in the intervention arm
Supplementary Table S2	Exploratory associations involving ethnic identity	Results 3.3	Exploratory correlations between identity change and psychological outcome change
Supplementary Table S 3	Reliability, floor, and ceiling dia gnostics	Methods/Results	Internal consistency and floor/ceiling s tatus of the main scales at each time p oint
Supplementary Table S 4	Data quality and response-style d iagnostics	Methods/Results	Missingness, straight-lining, noisy cas es, and quality-control handling
Supplementary Table S4a	Adjusted model audit for group-by-time contrasts	Methods/Results	Model-audit contrasts for adjusted group-by-time estimates
Supplementary Table S5	Exploratory heterogeneity screen	Discussion	Subgroup and heterogeneity screening, hypothesis-generating only
Supplementary Table S6	Interview participant characteristics	Results 3.4	Basic details of the T1 interview subsample
Supplementary Table S7	Qualitative themes and illustrative coding	Results 3.4	Themes, mention counts, and interpretive limits
Supplementary Table S8	Ryff sensitivity analyses	Methods/Results	Complete-case, per-protocol, and baseline-adjusted ANCOVA sensitivity checks for the primary Ryff outcome
Supplementary Figure S1	Exploratory ethnic identity pathway	Results/Discussion	Exploratory interpretive limits of the identity-related pathway
Supplementary File 1	De-identified intervention session framework	Methods	Replicable session structure for the integrated embroidery, storytelling, and group exchange program
Supplementary File 2	Semi-structured interview guide	Methods	Interview guide framework

Supplementary Table S1. Process evaluation and manipulation check results

Process evaluation item	n	Mean	SD	Min	Max
Clarity of cultural content	40	4.33	0.53	3.00	5.00
Rated importance of needlework	40	4.25	0.71	3.00	5.00
Rated importance of cultural content	40	4.33	0.57	3.00	5.00
Rated importance of group interaction	40	3.68	0.94	1.00	5.00
Perceived difference from ordinary handcraft	40	4.00	0.88	2.00	5.00
Perceived link to cultural identity	40	3.83	0.90	2.00	5.00
Overall process evaluation score	40	4.12	0.36	3.25	4.75

Note: All items were completed by the intervention group at T1. The table describes how participants perceived and rated the art's cultural character, the making process, and group interaction; absent component-separated control arms, it cannot be used to apportion their relative contributions.

Supplementary Table S2. Exploratory associations involving ethnic identity

Sample	Exploratory association	r	p value	n	Interpretation
Full analytic sample	T1 change in ethnic identity vs. T1 change in Ryff well-being	0.21	0.064	80	Exploratory association; no causal reading
Full analytic sample	T1 change in ethnic identity vs. T1 change in WEMWBS	-0.06	0.611	80	Exploratory association; no causal reading
Full analytic sample	Overall process score vs. T1 change in ethnic identity	0.19	0.238	40	Exploratory association; no causal reading
Intervention arm only	T1 change in ethnic identity vs. T1 change in Ryff well-being	0.16	0.331	40	Exploratory association; no causal reading
Intervention arm only	T1 change in ethnic identity vs. T1 change in WEMWBS	-0.19	0.239	40	Exploratory association; no causal reading
Intervention arm only	Overall process score vs. T1 change in ethnic identity	0.19	0.238	40	Exploratory association; no causal reading

Note: Identity correlations serve only to suggest possible mechanistic leads and cannot be read as mediation. They support further study of embroidery's cultural fit as a minority-arts vehicle, but cannot separate the contributions of cultural character, making, and group interaction.

Supplementary Table S3. Reliability, floor, and ceiling diagnostics

Scale	Time	n	Cronbach's alpha	Minimum corrected item-total r	Floor %	Ceiling %
Ryff well-being	T0	80	0.93	0.42	0.00	2.50
Ryff well-being	T1	76	0.91	0.44	0.00	0.00
Ryff well-being	T2	67	0.89	0.45	0.00	0.00
WEMWBS	T0	80	0.85	0.34	0.00	0.00
WEMWBS	T1	76	0.84	0.36	0.00	0.00
WEMWBS	T2	67	0.88	0.37	0.00	0.00
ULS-8 loneliness	T0	80	0.70	0.16	2.50	0.00
ULS-8 loneliness	T1	76	0.69	0.20	2.50	0.00
ULS-8 loneliness	T2	67	0.74	0.31	1.25	0.00
Social connectedness	T0	80	0.76	0.31	1.25	0.00
Social connectedness	T1	76	0.77	0.40	2.50	0.00
Social connectedness	T2	67	0.79	0.41	0.00	0.00
WHO-5	T0	80	0.77	0.44	1.25	1.25
WHO-5	T1	76	0.63	0.22	1.25	0.00
WHO-5	T2	67	0.69	0.32	0.00	1.25
Ethnic identity	T0	80	0.85	0.42	1.25	1.25
Ethnic identity	T1	76	0.83	0.32	0.00	1.25
Ethnic identity	T2	67	0.85	0.39	0.00	0.00

Note: The table gauges internal consistency and floor or ceiling risk. Main analyses used computable scale totals under the scoring rules, whereas T2 reliability and floor/ceiling diagnostics used participants with complete item-level data for these diagnostics (n = 67); this is why the diagnostic n is smaller than the 74 people who completed the T2 questionnaire. The low WHO-5 alpha at T1 warrants further reading against item behavior, scale version, and sample characteristics. Higher

ULS-8 scores mean greater loneliness; floor effects may reflect low baseline loneliness in some participants.

Supplementary Table S4. Data quality and response-style diagnostics

Domain	Diagnostic item	n (%)	Denominator / interpretation
Initial screening	Questionnaires returned	88	All returned questionnaires before quality scre ening
Initial screening	Excluded during baseline quality scr eening	8 (9.1%)	Denominator = 88; incomplete information, mi ssing linkage/timing, out-of-range responses, o r patterned responding
Initial screening	Valid baseline analytic sample	80 (90.9%)	Denominator = 88; retained for baseline and m ain longitudinal analyses where scale totals we re computable
Baseline quality review	Flagged by combined quality control for review	21 (26.3%)	Denominator = 80; review flag, not automatic exclusion
Baseline quality review	Flagged by student quality control	14 (17.5%)	Denominator = 80; subset of review flags
Baseline quality review	Straight-lining flagged for review	3 (3.8%)	Denominator = 80; same straight-lining cases, not an additional group
Baseline quality review	Noisy but retained after review	10 (12.5%)	Denominator = 80; retained to preserve realisti c response variability
T2 follow-up completion	Completed T2 questionnaire	74 (92.5%)	Denominator = 80; 37/40 in each group
T2 follow-up completion	Did not complete T2 questionnaire	6 (7.5%)	Denominator = 80
T2 non-completion reason	Lost to contact	3 (50.0%)	Denominator = 6 T2 non-completers
T2 non-completion reason	Scheduling conflict	2 (33.3%)	Denominator = 6 T2 non-completers
T2 non-completion reason	Health reason	1 (16.7%)	Denominator = 6 T2 non-completers
T2 item completeness am ong completers	No item missingness	63 (85.1%)	Denominator = 74 T2 completers
T2 item completeness am ong completers	Minor item missingness but scale tot als computable	11 (14.9%)	Denominator = 74 T2 completers; included in main scale-total analyses where scoring rules p ermitted
T2 item completeness am ong completers	Complete item-level data for reliabili ty/floor-ceiling diagnostics	67 (90.5%)	Denominator = 74 T2 completers; 67 = 63 with no item missingness plus 4 minor-missingness records still complete for these diagnostics; 7 minor-missingness records excluded from S3 d iagnostics.

Note: The table separates baseline screening, follow-up completion, T2 non-completion reasons, and item-level completeness so that each row has an explicit denominator. Review flags are not additive exclusion counts; cases could be flagged for review and still be retained after adjudication. The diagnostic n of 67 comprises 63 records with no item missingness on the tracked item set plus four records whose minor omissions did not affect the complete-item diagnostics used here; seven of the 11 records with minor missingness were excluded from S3 diagnostics, so 74 - 7 = 67.

Supplementary Table S4a. Adjusted model audit for group-by-time contrasts

Outcome	Contrast	Method	Model observations / pa rticipants	Adjusted estimate (95% CI)	p value
Ryff psychological well-b eing	group x T1	MixedLM	234 / 80	12.18 (6.28 to 18.09)	<0.001
Ryff psychological well-b eing	group x T2	MixedLM	234 / 80	6.22 (0.14 to 12.29)	0.045
WEMWBS well-being	group x T1	MixedLM	234 / 80	3.94 (0.62 to 7.25)	0.020
WEMWBS well-being	group x T2	MixedLM	234 / 80	0.90 (-2.51 to 4.31)	0.605
ULS-8 loneliness	group x T1	OLS fallback	234 / 80	-2.53 (-5.23 to 0.17)	0.067

Outcome	Contrast	Method	Model observations / pa rticipants	Adjusted estimate (95% CI)	p value
ULS-8 loneliness	group x T2	OLS fallback	234 / 80	-0.61 (-1.27 to 0.05)	0.072
Social connectedness	group x T1	MixedLM	234 / 80	2.66 (-0.11 to 5.43)	0.060
Social connectedness	group x T2	MixedLM	234 / 80	1.67 (-1.17 to 4.52)	0.250
WHO-5 well-being	group x T1	MixedLM	234 / 80	-0.59 (-2.62 to 1.44)	0.570
WHO-5 well-being	group x T2	MixedLM	234 / 80	1.69 (-0.39 to 3.78)	0.111
Ethnic cultural identity	group x T1	MixedLM	234 / 80	3.57 (0.68 to 6.46)	0.015
Ethnic cultural identity	group x T2	MixedLM	234 / 80	2.03 (-0.94 to 4.99)	0.181

Note: These contrasts use the corrected adjusted model in which education and income enter as categorical covariates, avoiding case deletion caused by ordinal recoding of valid response categories. The full fixed-effect coefficient table, including covariate terms, is exported as TableS4b_full_fixed_effect_coefficients.csv in the result package.

Supplementary Table S5. Exploratory heterogeneity screen

Screening variable	Level	Outcome	Between-group di fference	p value	Interpretation
Baseline Ryff tertile	Low	ULS-8	-4.79	0.011	Exploratory screen; sa mples too small for sub group conclusions
Baseline Ryff tertile	Middle	Ryff	12.41	0.017	Exploratory screen; sa mples too small for sub group conclusions
Baseline Ryff tertile	Middle	Cultural identity	5.55	0.012	Exploratory screen; sa mples too small for sub group conclusions
Baseline Ryff tertile	High	Ryff	14.92	0.046	Exploratory screen; sa mples too small for sub group conclusions
Baseline social contact	Low contact	Ryff	16.65	<0.001	Exploratory screen; sa mples too small for sub group conclusions
Baseline social contact	Low contact	Social connectedness	6.33	0.012	Exploratory screen; sa mples too small for sub group conclusions
Baseline social contact	Not low contact	Ryff	10.43	0.031	Exploratory screen; sa mples too small for sub group conclusions
Prior embroidery exper ience	None	Ryff	13.08	0.025	Exploratory screen; sa mples too small for sub group conclusions
Prior embroidery exper ience	Some prior	Ryff	11.08	0.011	Exploratory screen; sa mples too small for sub group conclusions
Prior embroidery exper ience	Some prior	Cultural identity	5.32	0.012	Exploratory screen; sa mples too small for sub group conclusions

Note: This exploratory heterogeneity screen covers baseline Ryff tertile, baseline social contact, and prior embroidery experience. With no multiplicity correction and small subgroups, it must not ground confirmatory subgroup claims.

Supplementary Table S6. Interview participant characteristics

Participant ID	Sex	Age	Retirement status	Prior embroidery experience	Attendance	Interview duration
I01	Female	59	Retired	Occasionally	8	31 min
I02	Male	61	Retired	Never	7	24 min
I03	Female	57	Near retirement	Often	8	29 min
I04	Female	63	Retired	Occasionally	6	22 min
I05	Male	60	Retired	Never	7	26 min
I06	Female	58	Near retirement	Never	8	32 min
I07	Female	62	Retired	Occasionally	5	18 min
I08	Male	64	Retired	Never	6	21 min
I09	Female	56	Near retirement	Often	8	30 min
I10	Female	65	Retired	Occasionally	7	25 min

Note: Basic characteristics of the semi-structured interview subsample drawn from the intervention arm at T1.

Supplementary Table S7. Qualitative themes and illustrative coding

Theme	Participants mentioning the theme	Illustrative initial codes	Interpretive role
Embroidery evokes cultural continuity and a felt closeness of identity	8/10	Childhood memory; women's experience in the family; Zhuang patterns; ethnic closeness	Helps explain the short-term T1 rise in ethnic identity
Finishing pieces brings mastery and daily rhythm	7/10	Something to do; small pieces completed; focus; sense of accomplishment	Helps explain the clearer immediate gain in psychological well-being
Group exchange offers companionship and mutual encouragement	6/10	Asking one another; attending together; chatting after class; less time cooped up at home	Helps explain favorable movement in loneliness and social connectedness
Storytelling sets the activity apart from plain handcraft, with varied individual experience	7/10	Knowing pattern meanings; more than passing time; eye strain; frustration at slow hands	Supports the cultural meaning of embroidery as a minority-arts vehicle while flagging implementation burden

Note: Mention counts describe content distribution only, not statistical tests. Qualitative findings interpret experience and acceptability and support the cultural fit of embroidery as a minority-arts vehicle; without component-separated arms, the relative contributions of cultural character, making, and interaction cannot be distinguished.

Supplementary Table S8. Ryff sensitivity analyses

Sensitivity check	Contrast	Analysis n	Estimate (intervention - waitlist)	95% CI	p value
Complete-case repeated-measures model	T1 group x time	74	12.79	6.70 to 18.89	<0.001
Complete-case repeated-measures model	T2 group x time	74	6.68	0.58 to 12.77	0.032

Sensitivity check	Contrast	Analysis n	Estimate (intervention - waitlist)	95% CI	p value
Per-protocol repeated-measures model	T1 group x time	75	12.34	6.33 to 18.35	<0.001
Per-protocol repeated-measures model	T2 group x time	75	7.00	0.91 to 13.10	0.024
Baseline-only ANCOVA	T1 Ryff	80	9.63	3.52 to 15.73	0.002
Fully adjusted ANCOVA	T1 Ryff	80	9.86	3.69 to 16.04	0.002
Fully adjusted + prior embroidery experience	T1 Ryff	80	10.25	3.79 to 16.72	0.002
Baseline-only ANCOVA	T2 Ryff	74	4.12	-1.18 to 9.41	0.125
Fully adjusted ANCOVA	T2 Ryff	74	4.43	-1.36 to 10.23	0.131
Fully adjusted + prior embroidery experience	T2 Ryff	74	4.33	-1.74 to 10.39	0.159

Note: Values are intervention-minus-waitlist estimates. Complete-case and per-protocol rows report group-by-time interaction contrasts from repeated-measures models for participants included in each sensitivity sample. ANCOVA rows report adjusted mean differences from models using post-baseline Ryff total scores as outcomes. Baseline-only ANCOVA models adjusted for baseline Ryff score. Fully adjusted ANCOVA models additionally included age, sex, education, living alone, income, and chronic disease; embroidery-experience models further included prior embroidery experience. These analyses were conducted as sensitivity checks for the primary Ryff outcome.

2 Supplementary Figure S1

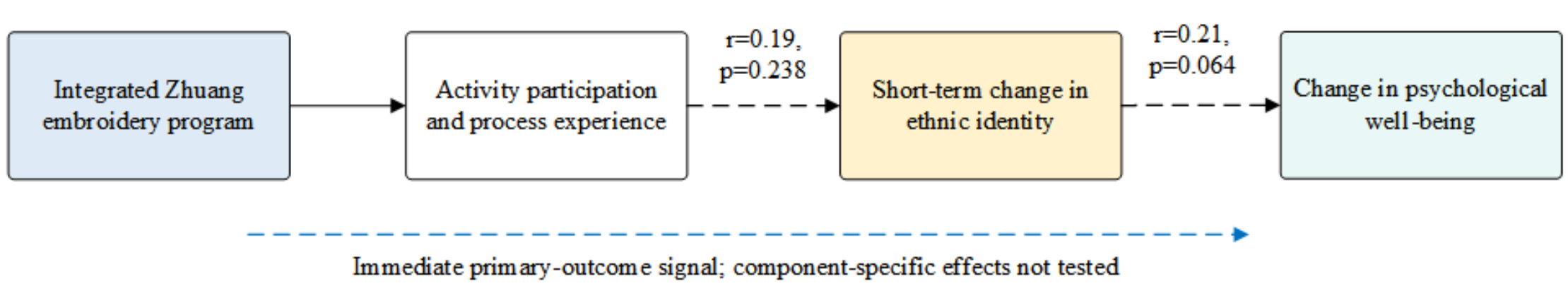

Supplementary Figure S1. Exploratory ethnic identity pathway

Note: The figure sketches only the exploratory interpretive framework running from the integrated program through activity participation and process experience to change in ethnic identity and, in turn, change in psychological well-being. Dashed paths mark hypothesized links unverified by the present design and should not be read as mediation.

3 **Supplementary File 1.** De-identified intervention session framework

The intervention was delivered once weekly for eight weeks, with each session lasting approximately two hours. The de-identified framework below summarizes the recurring session structure used to support fidelity while omitting community names, individual identifiers, and locally identifying examples.

**Session segment**	**Operational content**	**Implementation record**
Opening and attendance	Confirm attendance, prepare materials, and address brief questions from the previous se ssion.	Attendance log and protocol deviation note
Embroidery demonstration	Introduce the weekly embroidery task, stitch or pattern step, and expected achievable out put.	Delivery checklist and instructor record
Guided practice	Participants complete simplified or staged e mbroidery tasks with support from instructo rs and peer discussion.	Completion status, difficulties, and assistanc e recorded
Cultural storytelling	Provide a brief explanation of pattern meani ngs, traditional tales, local life memories, or cultural symbols linked to the session task.	Cultural content delivery recorded
Group exchange	Invite participants to share making experien ces, cultural recollections, reflections on fini shed pieces, and everyday changes noticed s ince participation.	Representative feedback and participation n otes
Closing record	Summarize completion, remind participants of the next session, and document any proto col deviations or practical barriers.	Completion record and deviation log

Note: This framework summarizes the recurring session structure rather than a verbatim script. The three components were delivered together and were not evaluated separately.

4 **Supplementary File 2. Semi-structured interview guide**

Semi-structured interviews were conducted at T1 with a subsample of the intervention arm to deepen understanding of participants' experience of embroidery practice, cultural storytelling, group exchange, and activity sustainability. The guide comprised the following questions:

1. What is your overall impression of the eight-week embroidery program?
2. Which parts left the deepest impression: the hands-on embroidery, the cultural storytelling, the group exchange, or something else?
3. Since taking part, have you noticed changes in your mood, daily routine, or sense of purpose in life?
4. Did the embroidery patterns, stories, or cultural talks bring to mind personal experiences, family memories, or Zhuang cultural experience?
5. Has learning and talking together in the group affected your connections with other people in the community?
6. After finishing pieces, did you feel accomplishment or mastery, and would you be willing to join similar activities again?
7. Now that the program has ended, which changes seem easiest to keep up, and which may be harder to maintain?
8. If such activities continue in future, what would you like added or adjusted?

Interviews proceeded only after recording consent was obtained, and transcripts were de-identified. Interview material served to interpret intervention experience and sustainability, not to replace the primary and secondary quantitative outcomes.
